# Be prepare to tackle Human Monkeypox: A real challenge for Bangladesh

**DOI:** 10.1002/hsr2.1022

**Published:** 2022-12-24

**Authors:** Ashis Talukder

**Affiliations:** ^1^ Statistics Discipline, Science Engineering and Technology School Khulna University Khulna Bangladesh

**Keywords:** Bangladesh, challenge, Human Monkeypox

The COVID‐19 outbreak in Bangladesh was the worst condition the country has ever seen, drastically impacting the whole healthcare system and seriously harming the general wellbeing of healthcare professionals. The entire nation was under lockdown, and COVID‐19 sufferers were clogging up healthcare facilities. Patients without COVID were simultaneously unable to get standard medical care. The worn‐out healthcare system was torn apart by the COVID‐19 epidemic, making it clear that urgent changes were required. In addition, the Mucormycosis and monkeypox viruses are now spreading throughout multiple nations.

Although monkeypox is a viral zoonosis, it is milder and rarely fatal than smallpox and belongs to the same virus family as the variola virus. Monkeypox has taken over as the most significant orthopoxvirus for public health following the eradication of smallpox in 1980 and consequent end to smallpox vaccination. Primarily affecting central and western Africa, monkeypox has been spreading into cities and is frequently seen close to tropical rainforests.^1^

The viral illness monkeypox was found to still be spreading in May 2022.^2^ The first case was found on May 6, 2022, in a person with connections to Nigeria through travel, and the earliest cluster of cases was found there.^3^ The outbreak marked the first significant expansion of monkeypox outside of Central and West Africa. Instances have been recorded as of May 18 from a growing number of countries and regions, notably in Europe but also in North and South America, Asia, Africa, and Oceania.^4^ The World Health Organization declared the pandemic a public health emergency of global significance on July 23. In all, 34,448 confirmed cases of monkeypox had been reported as of August 12 in 82 different nations, with the majority of those cases being the first reported in those nations.^5^

Despite the fact that no cases have been identified in Bangladesh to date, the nation has issued a health notice in an effort to contain the current monkeypox pandemic that has spread over the globe. The Daily Star (a popular newspaper) consulted with a number of eminent medical experts in Bangladesh to learn the best prevention strategies for the monkeypox virus. Since monkeypox enters the body through the nose, Prof. Dr. Nazrul Islam, a former vice chancellor of Bangabandhu Sheikh Mujib Medical University (BSMMU), advised applying the same rules, such as wearing a mask and frequently washing hands, to combat both coronavirus and monkeypox.^6^ Dr. Nazrul further stated that travelers at airports should be isolated for at least 14 days if they exhibit monkeypox symptoms.

Monkeypox requires 2–4 weeks to heal, according to Dr. ABM Abdullah, our prime minister's personal physician.^6^ He advised travelers to Bangladesh, especially those from Africa, to take precautions to stop the virus from spreading. He also emphasizes conducting in‐depth research into the diseases’ severity and symptoms.

Monkeypox disease is still not a major threat to Bangladesh as this country has not yet found any evidence of any monkeypox cases.^7^ However, according to the “Latest Educational, Business, Tech, Sarkari Result Updates” on December 8, 2022, India had ten confirmed instances of the disease, with one case each in Delhi and Telangana, four in Bihar, and four in Uttar Pradesh.^8^ Our medical professionals concentrated on raising knowledge of the monkeypox virus among the general public in light of this problem. Additionally, they recommended visiting the hospital if any symptoms appeared. Patients’ samples were to be collected by hospitals and sent to the IEDCR. A health screening at the airport was recommended for visitors to Bangladesh.

## AUTHOR CONTRIBUTIONS

**Ashis Talukder**: conceptualization; data curation; formal analysis; funding acquisition; investigation; methodology; project administration; resources; software; supervision; validation; visualization; writing—original draft; writing—review & editing.

## CONFLICT OF INTEREST

The author declares no conflict of interest.

## TRANSPARENCY STATEMENT

The lead author (**Ashis Talukder**) affirms that this manuscript is an honest, accurate, and transparent account of the study being reported; that no important aspects of the study have been omitted; and that any discrepancies from the study as planned (and, if relevant, registered) have been explained.

## Data Availability

Data sharing not applicable to this article as no datasets were generated or analysed during the current study.
